# Is seborrheic dermatitis associated with early-stage osteoarthritis?

**DOI:** 10.1097/MD.0000000000037217

**Published:** 2024-02-09

**Authors:** Sevgi Kulakli, Fazil Kulakli, Betül Yilmaz, İlker Fatih Sari, Işil Deniz Oğuz

**Affiliations:** a Giresun University Faculty of Medicine, Department of Dermatology and Venereology, Giresun, Turkey; b Giresun University Faculty of Medicine, Department of Physical Medicine and Rehabilitation, Giresun, Turkey.

**Keywords:** cartilage thickness, femoral cartilage, osteoarthritis, seborrheic dermatitis, ultrasound

## Abstract

Seborrheic dermatitis (SD) and osteoarthritis involve similar factors in their pathogenesis. Both of these diseases are associated with an increased frequency of metabolic syndrome and underlying systemic inflammation. This study evaluated the thickness of the distal femoral cartilage using ultrasonography in patients with SD. The study enrolled 60 patients with SD (19 females and 41 males, mean age: 34.07 ± 12.56 years) and 60 controls matched for age and sex (20 females and 40 males, mean age: 35.08 ± 12.78 years). Ultrasonography was used to measure the distal femoral cartilage thickness (FCT) of the right medial condyle, right lateral condyle, right intercondylar area, left medial condyle, left lateral condyle, and left intercondylar area. FCT values at all points were significantly higher in patients with SD than in the controls (*P* < .05). Further, all FCT values were significantly higher in patients with moderate SD than in those with mild SD (*P* < .001). A strong positive correlation was observed between disease severity and FCT measured at right medial condyle (r = .7, *P* < .001), right lateral condyle (r = .749, *P < *.001), right intercondylar area (r = .79, *P < *.001), left medial condyle (r = .624, *P* < .001), and left intercondylar area (r = .703, *P < *.001). Further, a moderately positive correlation was observed between disease severity and FCT measured at left lateral condyle (r = .581, *P < *.001). Increased FCT in patients with SD might be an early indicator of osteoarthritis. However, further studies, especially those evaluating older patients with SD, are required to support our findings.

## 1. Introduction

Seborrheic dermatitis (SD), a common inflammatory cutaneous disease, is characterized by chronic, recurrent erythematous and scaly plaques in sebaceous areas such as the scalp, face, presternal area, and interscapular region. Its prevalence is approximately 5% in the general population, and it particularly affects adolescents and young adults.^[1]^ The pathogenesis of SD remains unknown and may involve *Malassezia* and *Demodex* species, certain medications, ultraviolet radiation, androgens, various environmental factors, and nutritional factors. Notably, the higher prevalence in males and its onset during adolescence implies that androgens, which stimulate the sebaceous glands, play a significant role in the etiology of SD.^[2]^ The increased frequency of SD in individuals with acquired immune deficiency syndrome, lymphoma, and bone marrow suppression suggests a significant role of the immune system in its pathophysiology. High levels of numerous inflammatory cytokines, including interleukin (IL)-1α, IL-1ß, IL-2, IL-4, IL-6, IL-8, IL-10, IL-12, IL-17, and tumor necrosis factor (TNF)-α, have been reported in patients with SD.^[3]^ Further, increased levels of inflammatory markers, along with an elevated frequency of metabolic syndrome and osteoporosis, suggest the presence of subclinical systemic inflammation in patients with SD.^[4–9]^

Osteoarthritis (OA) is a common musculoskeletal disorder that affects all joint structures, primarily the cartilage. This phenomenon is most commonly observed in knee joints. Factors such as advanced age, female sex, obesity, trauma, and genetic predisposition are crucial contributors to OA development and progression. In normal joint cartilage, a balance exists between the formation and breakdown of the structures that comprise the extracellular matrix. However, this balance is disrupted in OA, leading to the increased destruction of these structures.^[10,11]^ OA pathogenesis begins with an increase in proinflammatory cytokines, resulting in the activation of various catabolic enzymes, especially matrix metalloproteinases, in the intra-articular space, leading to cartilage damage. Major cytokines involved in the pathogenesis include IL-1ß, IL-6, IL-17, IL-18, IL-21, IL-22, and TNF-ɑ.^[12,13]^ Additionally, systemic metabolic inflammation and metabolic syndrome are more commonly observed in individuals with OA.^[14]^

To date, no studies have evaluated the relationship between SD and OA in the English literature. This study aimed to investigate the relationship between these 2 diseases, in which similar factors play a role in pathogenesis.

## 2. Methods

The study included patients aged > 18 years diagnosed with SD in the dermatology outpatient clinic. Pregnant women; patients with endocrine, renal, hepatic, or rheumatic diseases; malignancies; those taking any medication affecting skeletal metabolism (such as systemic steroids, heparin, and anticonvulsants); or those with a history of trauma or surgery of the knee were excluded from the study. All patients were evaluated by the same dermatologists, and their age, sex, occupation, family history, duration of SD, smoking status, exercise habits, height, weight, body mass index (BMI), and dermatological examination findings were recorded. Disease severity was determined based on the Seborrheic Dermatitis Area and Severity Index (SDASI) score, which is calculated by scoring erythema, scaling, and itching for each affected area on a scale of 0 to 3, where 0 represents none, 1 represents mild, 2 represents moderate, and 3 represents severe. The score obtained for each area was then multiplied by the specific coefficient assigned to that area. The total score was calculated using the following formula: scalp (0.1) + hair-bearing skin (0.4) + nasolabial area (0.1) + eyebrows (0.1) + postauricular area (0.1) + ears (0.1) + mid-chest (0.2) + back (0.2) + cheeks and chin (0.1).^[15]^ The patients were classified as having a mild disease if their SDASI score was 0 to 4.4, moderate disease if 4.5 to 8.5, and severe disease if 8.6 to 12.6. Patients who engaged in moderate-to-vigorous exercise for at least 30 minutes daily or at least 3 days a week were considered to be actively exercising.

The control group comprised healthy adults matched for age and sex with the study group who were not pregnant, did not have endocrine, renal, hepatic, or rheumatic diseases, malignancies, or were not taking any medication affecting skeletal metabolism, and had no history of knee trauma or surgery.

**Figure 1.**
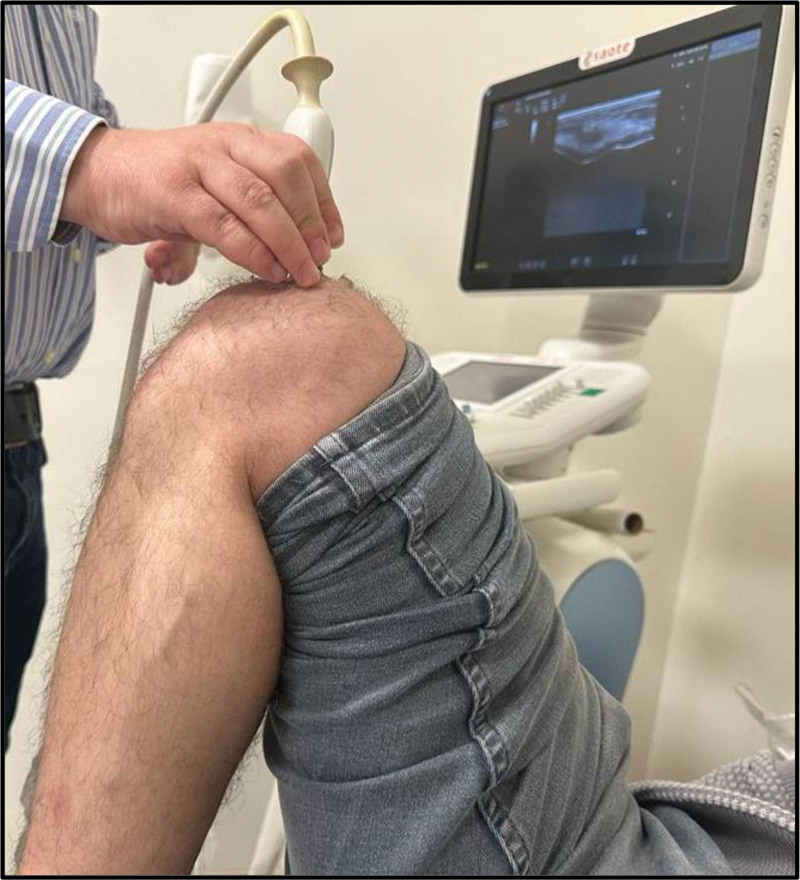
Position of the probe and the patient during ultrasonographic evaluation.

**Figure 2.**
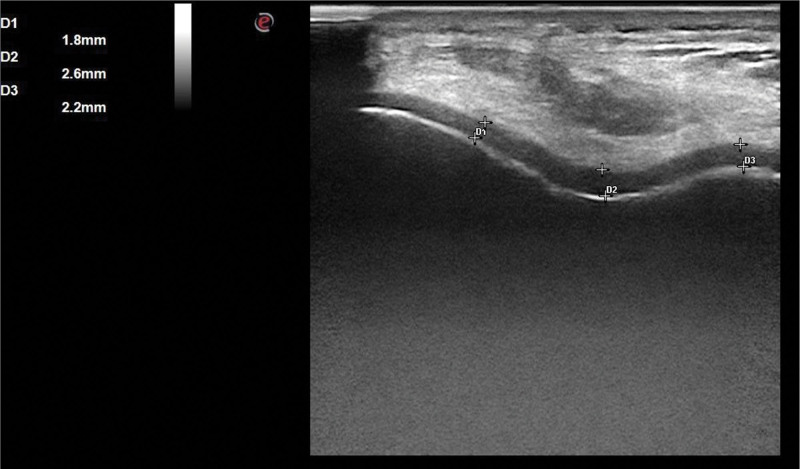
Ultrasonografic measurement of right distal femoral cartilage thickness. D1, right lateral condyle; D2, right intercondylar area; D3, right medial condyle.

All participants were evaluated by a physical medicine and rehabilitation physician who was blind to the participants’ conditions for knee pain and morning stiffness. A knee examination, including crepitation and widening of the knee joint, was performed. Another physician experienced in musculoskeletal system ultrasonography (US) and blinded to the clinical status of the participants measured the distal femoral cartilage thickness (FCT) using US. Ultrasonographic examination was performed with a 1 to 22 MHz linear ultrasound probe (MyLabSix, Esoata Group, Genoa, Italy) by positioning it axially over the suprapatellar region in patients in a supine position with their knees maximally flexed (Fig. 1). Distal FCT measurements were performed at specific anatomical points: the central regions of the right medial condyle (RMC), right lateral condyle (RLC), right intercondylar area (RIC), left medial condyle (LMC), left lateral condyle (LLC), and left intercondylar area (LIC). This process involved determining the distance between 2 clearly defined hyperechoic lines at the synovial space-cartilage and cartilage-bone interfaces (Fig. 2).

### 2.1. Statistical analysis

Data were analyzed using IBM SPSS software (version 23.0; SPSS, Chicago, IL). Numerical data were presented as mean ± standard deviation or median, based on the normal distribution of descriptive statistical data. Categorical data were expressed as frequencies and percentages. To compare numerical data between the 2 groups, an independent sample *t*-test was used if the assumption of normality was met; otherwise, the Mann–Whitney *U* test was employed. Accordingly, the chi-square or Fisher exact tests were used to compare categorical data between the 2 groups. The Spearman correlation test was used to evaluate the correlation between disease duration and severity and FCT because of their non-normal distribution in patients with SD. Additionally, for the relationship between SDASI scores and cartilage thickness, a linear regression analysis was performed and a scatter plot was constructed. Statistical significance was set at *P < *.05.

## 3. Results

The study comprised 60 patients with SD, with a mean age of 34.07 ± 12.56 years (19 females and 41 males), and 60 controls with a mean age of 35.08 ± 12.78 years (20 females and 40 males). The 2 groups were comparable in terms of age and sex (*P = *.603 and *P = *.845, respectively). The mean duration of SD was 5.26 ± 6.7 years. The most common lesion locations were the scalp and face (48.3%). Forty-two (70%) patients had mild SD, 18 (30%) had moderate SD, and none had severe SD. Among the patients, 12 (20%), 1 (1.7%), and 3 (5%) had bilateral knee pain, morning stiffness, and crepitations, respectively. Table 1 shows the demographic and clinical characteristics of the patients.

**Table 1:** Demographic and clinical characteristics of patients with seborrheic dermatitis.

	Patients (n = 60)
Age (years), mean ± standard deviation	34.07 ± 12.56
Sex, n (%) Female Male	19 (31.7) 41 (68.3)
Disease duration (years), mean ± standard deviation	5.26 ± 6.7
Severity, n (%) Mild Moderate	42 (70) 18 (30)
Localization, n (%) Skalp Face Skalp and face Skalp and ears Skalp, face, trunk Skalp, face, ears Skalp, face, ears, trunk	8 (13.3) 2 (3.3) 29 (48.3) 5 (8.3) 7 (11.7) 6 (10) 3 (5)
Family history of seborrheic dermatitis, n (%) Present Absent	19 (31.7) 41 (68.3)
Knee joint symptoms and signs, n (%) Pain Morning stiffness Crepitation Widening	12 (20) 1 (1.7) 3 (5) 0 (0)

No noteworthy differences in height, weight, BMI, smoking status, exercise habits, or occupation were observed between patients with SD and controls (*P > *.05). However, pain in the right and left knee was significantly more frequent in patients with SD than the controls (*P = *.032). In addition, no statistically significant differences in morning stiffness, crepitations, or knee widening were observed between the 2 groups. FCT was significantly greater in patients with SD than in the control group at all measurement points (RMC, RLC, RIC, LMC, LLC, and LIC) (*P < *.05). The demographic characteristics and FCT values of the patients and controls are shown in Table 2. The FCT value was higher in patients with moderate SD compared with those with mild SD (*P < *.001) (Table 3).

**Table 2:** Demographic characteristics and femoral cartilage thickness measurements of all subjects.

	Patients (n = 60)	Control (n = 60)	*P*
Age (years), mean (IR)	31.5 (19.5)	33.5 (17.75)	.603‡
Sex, n (%) Female Male	19 (31.7) 41 (68.3)	20 (33.3) 40 (66.7)	.845§
Height (m), mean ± SD	1.71 ± 0.83	1.70 ± 0.89	.745†
Weight (kg), mean ± SD	78.86 ± 13.66	75.41 ± 14.67	.184†
BMI (kg/m^2^), mean ± SD	26.9 ± 4.21	25.84 ± 4.37	.183†
Smoking, n (%) Smoker and ex-smoker Never smoked	16 (26.7) 44 (73.3)	17 (28.3) 43 (71.7)	.838§
Regular exercise, n (%) Present Absent	3 (5) 57 (95)	6 (10) 54 (90)	.491¶
Occupation, n (%) Student Blue collar Housewife White collar Retired	12 (20) 17 (28.3) 14 (23.3) 13 (21.7) 4 (6.7)	14 (23.3) 9 (15) 8 (13.3) 25 (41.7) 4 (6.7)	.086¶
Right knee, n (%) Pain	12 (20)	4 (6.67)	**0.032** §
Morning stiffness	1 (1.67)	0 (0)	>.99¶
Crepitation	3 (5)	1 (1.67)	.619¶
Widening	0 (0)	0 (0)	–
Left knee, n (%) Pain	12 (20)	4 (6.67)	**.032** §
Morning stiffness	1 (1.67)	0 (0)	>.99¶
Crepitation	3 (5)	1 (1.67)	.619¶
Widening	0 (0)	0 (0)	–
RMC (mm), mean ± SD	2.33 ± 0.43	2.13 ± 0.4	**.009** †
RLC (mm), mean ± SD	2.29 ± 0.43	2.11 ± 0.35	**.012** †
RIC (mm), median (IR)	2.7 (0.7)	2.1 (0.7)	**<.001** ‡
LMC (mm), mean ± SD	2.34 ± 0.41	2.11 ± 0.38	**.003** †
LLC (mm), median (IR)	2.35 (0.68)	2 (0.58)	**.003** ‡
LIC (mm), median (IR)	2.5 (0.8)	2.25 (0.58)	**.001** ‡

BMI = body mass index, IR = interquartile range, LIC = left intercondylar area, LLC = left lateral condyle, LMC = left medial condyle, RIC = right intercondylar area, RLC = right lateral condyle, RMC = right medial condyle, SD = standard deviation.

† Independent samples *t* test.

‡ Mann-Whitney U test.

§ Pearson chi-square test.

¶ Fisher exact test. Bold values, *P* < .05.

**Table 3:** Comparison of demographic, clinical characteristics, and femoral cartilage thickness in patients with mild and moderate seborrheic dermatitis.

	Mild	Moderate	*P*
Age (years), mean ± SD	33.5 ± 11.27	35.38 ± 15.43	.598†
Sex, n (%) Female Male	16 (38.1) 26 (61.9)	3 (16.7) 15 (83.3)	.135§
Height (m), mean ± SD	1.70 ± 0.83	1.72 ± 0.86	.445†
Weight (kg), mean ± SD	79.47 ± 12.68	77.44 ± 16.02	.602†
BMI (kg/m^2^), mean ± SD	27.26 ± 3.84	26.01 ± 4.9	.295†
Smoking,n (%) Smoker and ex-smoker Never smoked	13 (31) 29 (69)	3 (16.7) 15 (83.3)	.346§
Regular exercise, n (%) Present Absent	3 (7.1) 39 (92.9)	0 (0) 18 (100)	.547¶
Occupation, n (%) Student Blue collar Housewife White collar Retired	8 (19) 10 (23.8) 12 (28.6) 11 (26.2) 1 (2.4)	4 (22.2) 7 (38.9) 2 (11.1) 2 (11.1) 3 (16.7)	.102¶
Disease duration (years), median (IR)	2 (4)	2.5 (7.5)	.357‡
RMC (mm), mean ± SD	2.16 ± 0.37	2.72 ± 0.27	**<.001** †
RLC (mm), mean ± SD	2.13 ± 0.38	2.67 ± 0.29	**<.001** †
RIC (mm), mean ± SD	2.42 ± 0.43	3.15 ± 0.39	**<.001** †
LMC (mm), mean ± SD	2.17 ± 0.37	2.71 ± 0.2	**<.001** †
LLC (mm), mean ± SD	2.23 ± 0.4	2.69 ± 0.38	**<.001** †
LIC (mm), mean ± SD	2.36 ± 0.5	2.98 ± 0.33	**<.001** †

BMI = body mass index, IR = interquartile range, LIC = left intercondylar area, LLC = left lateral condyle, LMC = left medial condyle, RIC = right intercondylar area, RLC = right lateral condyle, RMC = right medial condyle, SD = standard deviation.

† Independent samples *t*-test.

‡ Mann–Whitney *U* test.

§ Pearson chi-square test.

¶ Fisher exact test. Bold values, *P* < .05.

**Figure 3.**
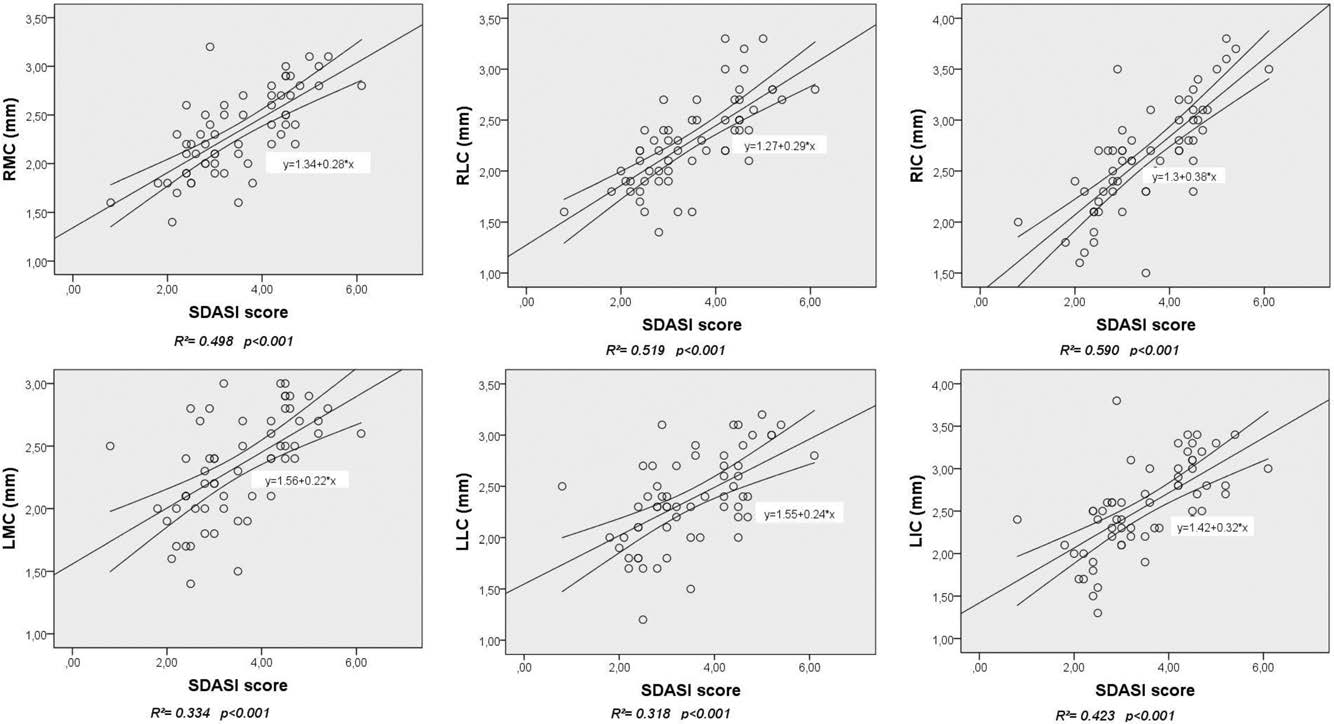
Relationship between Seborrheic Dermatitis Area and Severity Index (SDASI) scores and cartilage thickness with scatter plot.

A strong positive correlation was observed between disease severity and FCT measured at RMC (r = .7, *P < *.001), RLC (r = .749, *P < *.001), RIC (r = .79, *P* < .001), LMC (r = .624, *P < *.001), and LIC (r = .703, *P < *.001). Further, a moderately positive correlation was observed between disease severity and FCT measured at LLC (r = .581, *P < *.001). Disease duration exhibited a low or insignificant positive correlation only with the FCT measured at RLC (r = .269, *P = *.038). No correlation was identified between the thickness of other regions and disease duration (*P > *.05) (Table 4). Additionally, a linear regression analysis was conducted to explore a potential relationship between SDASI scores and cartilage thickness. Figure 3 shows a scatter plot of the SDASI scores on the cartilage thickness regression line and 95% confidence interval. A statistically significant relationship was observed between the SDASI scores and RMC (R2 = .498, *P < *.001), RLC (R2 = .519, *P < *.001), RIC (R2 = .590, *P < *.001), LMC (R2 = .334, *P < *.001), LLC (R2 = .318, *P < *.001), and LIC (R2 = .423, *P < *.001).

**Table 4:** The correlation between disease severity and duration with femoral cartilage thickness values.

	RMC	RLC	RIC	LMC	LLC	LIC
Disease duration r *P*	.127 .332	.269* .038	.204 .117	−.054 .682	.066 .614	.098 .455
Disease severity r *P*	.7** <.001	.749** <.001	.79** <.001	.624** <.001	.581** <.001	.703** <.001

Spearman test was used for correlation analysis.

RMC = right medial condyle; RLC = right lateral condyle; RIC = right intercondylar area; LMC = left medial condyle; LLC = left lateral condyle; LIC = left intercondylar area; r = Rho correlation coefficient.

* Correlation is significant at the.05 level. Bold values, *P* < .05.

** Correlation is significant at the.01 level.

## 4. Discussion

SD is a common, chronic, inflammatory, and recurrent skin disease that primarily affects the seborrheic areas.^[3]^ Notably, chronic inflammatory skin diseases, including psoriasis, rosacea, lichen planus, and hidradenitis suppurativa, may be accompanied by comorbidities such as metabolic syndrome, diabetes mellitus, obesity, and cardiovascular diseases.^[7]^ SD has been associated with insulin resistance, hypertension, dyslipidemia, and increased inflammatory parameters.^[4,5,7–9]^ OA is a chronic disease characterized by early-stage edema and swelling of the cartilage, particularly in weight-bearing joints such as the knee. The later stages of OA are characterized by cartilage loss and changes in the subchondral bone. OA and SD are prevalent worldwide and exhibit common etiopathogenic factors.^[3–6,16]^ Therefore, a relationship may exist between them. The assessment of femoral cartilage structure and thickness is a widely accepted method for the evaluation of knee OA.^[16–18]^ In this study, we used US because of its cost-effectiveness, ease of application, ability to perform dynamic measurements, and effectiveness in evaluating the distal cartilage structure of the femur.^[17,18]^ Notably, we observed a statistically significant increase in the distal FCT among patients with SD compared with controls.

OA progression involves an initial change in the cartilage matrix, which is characterized by an increase in the water content either before or during fibrillation. In the early stages, soft and swollen cartilage because of increased hydration can be observed macroscopically. This is attributed to the loss of the ability of the relaxed collagen network to resist the osmotic load created by aggrecan molecules. Although proteoglycan concentration increases initially, it decreases as the disease progresses, and the length of the glycosaminoglycan chains shortens. The reduction in keratan sulfate is accompanied by an increase in the ratio of chondroitin 4 sulfate to chondroitin 6 sulfate. Once the loss of proteoglycans reaches a certain threshold, the initially elevated water content decreases to below-normal levels.^[19–23]^ Hosseininia et al reported that augmented cartilage water content in hip OA is linked to OA pathology and can be detected in the early stages of OA.^[24]^ In addition, an association has been reported between FCT measured using US and macroscopically evaluated cartilage thickness. The ultrasonographic measurements were similar to the histological measurements of joint cartilage thickness.^[25–27]^Further, Öztürk et al, in their study evaluating FCT in individuals with pes planus, reported increased FCT, suggesting that this may be related to cartilage swelling occurring in the early stages of OA.^[17]^ Calvo et al, in their studies on rabbits with experimental knee OA, observed swelling in the knee cartilage in the early stages of OA through magnetic resonance imaging (MRI). They suggested that an increase in hyaline cartilage height could indicate tissue deterioration in the earliest stages of OA.^[28,29]^

Recently, metabolic inflammation has been more commonly observed in patients with SD and OA. Systemic inflammation, a risk factor for OA, may contribute to the risk of developing OA in patients with SD.^[14]^ Apparently, inflammatory cytokines such as IL-1ß, IL-6, IL-17, and TNF-ɑ commonly contribute to the etiopathogenesis of both SD and OA,^[4,6,12–14]^ leading to increased FCT, an early sign of OA, in patients with SD.

Androgens play a significant role in the pathogenesis of SD, as indicated by the higher prevalence of SD in males, which is more frequent in the first 3 months of life when sebaceous glands are more active due to the influence of maternal androgens and their onset during adolescence.^[2,20]^ In a study evaluating the relationship between skin diseases and hormone levels in patients with polycystic ovary syndrome (PCOS), a condition characterized by hyperandrogenism, SD was observed in 52.5% of the patients with PCOS, and seborrhea was associated with elevated levels of free testosterone and dehydroepiandrosterone sulfate.^[30]^ Another study found that the second-to-fourth-digit ratio, which is considered a marker of prenatal androgen exposure, was lower in males with SD than in controls.^[31]^ The 1.5-times higher OA prevalence in women than in men, which particularly becomes symptomatic during the postmenopausal period, indicates the role of hormonal factors in its pathogenesis.^[32]^ Notably, the cartilage volume, a helpful and sensitive tool for evaluating knee OA, is higher in men than in women.^[33]^ In an MRI study of asymptomatic males, a positive relationship was reported between testosterone levels and tibial cartilage volume in the medial region; however, in a longitudinal study over a 2-year period, a positive association was observed between testosterone levels and the loss of tibial cartilage volume.^[33,34]^ In an in vivo study conducted by Ma et al that compared intact male mice with those undergoing orchiectomy, intact mice exhibited more severe OA. Furthermore, orchiectomized male mice supplemented with dihydrotestosterone developed severe OA.^[35]^ In 3 different studies assessing FCT in patients with PCOS, FCT was significantly greater in patients with PCOS than in age- and BMI-matched healthy controls.^[36–38]^ Despite cartilage thinning or loss being a pathognomonic feature of OA, increased cartilage thickness in young adults may indicate tissue deterioration during the earliest stages of OA.^[38]^

The initial change in the development of OA is an increase in cartilage water content, leading to edema. The participants of our study comprised a young age group without any risk factors for OA. Therefore, the increased cartilage thickness observed in the patients with SD may be associated with an increased frequency of early-stage OA. Furthermore, we found that as the severity of the disease increased, cartilage thickness was also increased. Therefore, we hypothesize that individuals with SD may be at a higher risk of developing OA, and that as the severity of the disease increases, the severity of OA may also increase.

Exercise and weight control are strongly recommended in all guidelines for the management of OA.^[39,40]^ Notably, metabolic syndrome and obesity are more common in both patients with SD and OA.^[4,6,14,39]^ Therefore, exercise and weight control should be integrated into the routine follow-up of patients with SD. We believe that the risk of OA as well as metabolic syndrome may decrease in patients with SD owing to the anti-inflammatory effects of exercise and diet.^[41,42]^

The main limitations of our study include the lack of direct radiography or MRI for OA assessment, lack of functional assessment of the knee, lack of evaluation of other joints such as the hip or hand joints, lack of an assessment of sex hormone levels, and absence of patients with severe SD.

We believe that the observed increase in FCT evaluated using US in patients with SD may be an early indicator of OA. Considering that OA can significantly reduce the quality of life in these patients and lead to serious morbidity, we emphasize the importance of incorporating exercise and weight control into the routine follow-up of patients with SD to prevent OA development. Notably, our study is the first to evaluate this aspect, and further advanced studies, especially those involving older patients with SD and assessing their sex hormone profiles, are required for a comprehensive understanding of this topic.

## Author contributions

**Conceptualization:** Sevgi Kulakli, Fazil Kulakli.

**Data curation:** Sevgi Kulakli, Fazil Kulakli, Betül Yilmaz, Işil Deniz Oğuz.

**Formal analysis:** Sevgi Kulakli, İlker Fatih Sari.

**Investigation:** Sevgi Kulakli, Fazil Kulakli, Betül Yilmaz, İlker Fatih Sari, Işil Deniz Oğuz.

**Methodology:** Sevgi Kulakli, Fazil Kulakli, İlker Fatih Sari.

**Project administration:** Sevgi Kulakli, Fazil Kulakli, Betül Yilmaz, İlker Fatih Sari, Işil Deniz Oğuz.

**Resources:** Sevgi Kulakli.

**Software:** Sevgi Kulakli, İlker Fatih Sari.

**Supervision:** İlker Fatih Sari, Işil Deniz Oğuz.

**Validation:** Sevgi Kulakli, Fazil Kulakli.

**Visualization:** İlker Fatih Sari, Işil Deniz Oğuz.

**Writing – original draft:** Sevgi Kulakli.

**Writing – review & editing:** Fazil Kulakli.
